# Peer Review of “Impact of the COVID-19 Pandemic on Latino Families With Alzheimer Disease and Related Dementias: Qualitative Interviews With Family Caregivers and Primary Care Providers”

**DOI:** 10.2196/57160

**Published:** 2024-03-08

**Authors:** Anonymous

**Keywords:** Latino, dementia, caregiving, COVID-19, Alzheimer’s disease, health disparity, qualitative research, transcript analysis, health inequality, minority population, epidemiology, peer support, social services, health care, Alzheimer’s, minority, qualitative, interview, caregiver, primary care, impact, resilience, disparity, outcome, Alzheimer disease, Alzheimer

*This is the peer-review report for “Impact of the COVID-19 Pandemic on Latino Families With Alzheimer Disease and Related Dementias: Qualitative Interviews With Family Caregivers and Primary Care Providers*.”

## Round 1 Review

### General Comments

This paper [1] describes a qualitative study in which the authors seek to understand the experiences of Latino families managing Alzheimer disease and related dementias (ADRD). The authors interviewed both family caregivers and primary care providers (PCPs). This is a well-written manuscript that focuses on caregiving during COVID-19, a relatively understudied area. The authors note that this study represents secondary analyses of a larger study that focused on improving ADRD care services in primary care across settings.

### Specific Comments

#### Major Comments

1. My key methodological critique is that the authors should follow the COREQ (Consolidated Criteria for Reporting Qualitative Research) recommendations in reporting this study. Since this is a completed study, it is possible that the authors will not meet all the criteria; however, it is still important to know which recommendations were followed and which were not.
2. My key conceptual critique is that the authors do not provide any rationale as to why family caregivers’ and PCPs’ perspectives are included together. While I certainly understand that these are secondary analyses, the study introduction still needs to justify why these 2 stakeholder groups will provide perspectives that can be synthesized.
3. Relatedly, the authors should provide a conceptual or theoretical framework that guided this study.
4. In methods, please clarify whether the main interview was 45-60 minutes and the additional COVID-19–specific questions were excluded from this time. Please also provide the interview guide as an appendix (see COREQ) and include sample questions in the manuscript.
5. Please clarify what is meant by “the interviewers emphasized that participants were the experts to reduce power differentials.” How was this accomplished? How did the interviewer ensure that this power differential existed and that it was subsequently reduced?
6. Consider expanding the description of theme 1 to capture the dimensions of impact noted in the subthemes, for instance, “Both caregivers and PCPs highlighted the physical, psychological and social impacts of the pandemic on patients with ADRD.”
7. The second theme does not appear to integrate the PCP and caregiver views as well as theme 1. Consider splitting the current theme 2 into 2: theme 2 could be about individual coping and resilience, and theme 3 could be about systems-level factors, which would include vaccination acceptance and remote communications.
8. The conclusion discusses death and formal care, which are not highlighted in the themes or results. Anchor the discussion to the results of this study.

#### Minor Comments

1. Please review and correct typographical errors; for example, in data analysis, it says “fist author” instead of “first author.”
2. The citation #16 is for focus groups, but the authors did 1:1 interviews. Please ensure that this citation is correct.
3. The percentages in Table 1 are not meaningful given the small sample size. Please report only the Ns.

## Abbreviations

ADRD: Alzheimer disease and related dementias
COREQ: Consolidated Criteria for Reporting Qualitative Research
PCP: primary care provider
